# Accelerating DNA computing via freeze-thaw cycling

**DOI:** 10.1126/sciadv.aax7983

**Published:** 2023-08-25

**Authors:** Yun Zhu, Xiewei Xiong, Mengyao Cao, Li Li, Chunhai Fan, Hao Pei

**Affiliations:** ^1^ State Key Laboratory of Molecular and Process Engineering, Shanghai Key Laboratory of Green Chemistry and Chemical Processes, Shanghai Frontiers Science Center of Molecule Intelligent Syntheses, School of Chemistry and Molecular Engineering, East China Normal University, 500 Dongchuan Road, Shanghai 200241, China.; ^2^ School of Chemistry and Chemical Engineering, New Cornerstone Science Laboratory, Frontiers Science Center for Transformative Molecules and National Center for Translational Medicine, Shanghai Jiao Tong University, Shanghai 200240, China.; ^3^ Institute of Molecular Medicine, Shanghai Key Laboratory for Nucleic Acids Chemistry and Nanomedicine, Renji Hospital, School of Medicine, Shanghai Jiao Tong University, Shanghai 200127, China.

## Abstract

DNA computing harnesses the immense potential of DNA molecules to enable sophisticated and transformative computational processes but is hindered by low computing speed. Here, we propose freeze-thaw cycling as a simple yet powerful method for high-speed DNA computing without complex procedures. Through iterative cycles, we achieve a substantial 20-fold speed enhancement in basic strand displacement reactions. This acceleration arises from the utilization of eutectic ice phase as a medium, temporarily increasing the effective local concentration of molecules during each cycle. In addition, the acceleration effect follows the Hofmeister series, where kosmotropic anions such as sulfate (SO_4_^2−^) reduce eutectic phase volume, leading to a more notable enhancement in strand displacement reaction rates. Leveraging this phenomenon, freeze-thaw cycling demonstrates its generalizability for high-speed DNA computing across various circuit sizes, achieving up to a remarkable 120-fold enhancement in reaction rates. We envision its potential to revolutionize molecular computing and expand computational applications in diverse fields.

## Teaser

Freeze-thaw cycling is employed as a straightforward and versatile method for high-speed DNA computing on a large scale.

## INTRODUCTION

Molecular-engineered systems have demonstrated the ability to reliably perform computations, from signal transmission to information processing, which opens up the possibility of embedding control and functionality within complex molecular environments (*1*–*5*). Adleman’s pioneering work (*6*) demonstrated that DNA can serve as a powerful computing substrate. DNA sequence can encode algorithms to perform the operations of the computation that can solve Hamiltonian path problems, thus paving the way for DNA computing. The systematic use of DNA strand displacement pioneered by Yurke *et al.* (*7*) enabled algorithms to be encoded in DNA reaction networks (*8*). This approach offers simple primitives to construct sophisticated DNA computers with autonomous dynamic behavior, including digital logic circuits (*9*–*11*), catalytic networks (*8*, *12*, *13*), DNA oscillators (*14*), and neural networks (*15*–*17*). For instance, using DNA strand displacement reactions as the main computation mechanism, researchers have constructed multilayer logic circuits that can compute Boolean logic and a four-bit square-root function (*10*). DNA strand displacement circuitry has also been used to create neural networks that can classify sophisticated patterns of molecular events (*15*, *16*), such as handwritten digits. Recently, researchers demonstrated a large DNA strand displacement circuit with over 500 rationally designed DNA strands to implement convolutional neural network (ConvNet) computation (*17*), which enables 32-pattern recognition. These results suggest that DNA-based circuits have made great progress in scalability and power, and this “DNA on DNA” computation still holds great potential for further scaling up the complexity of DNA circuits. However, these circuits are based on the random collision and interaction between freely diffusing DNA molecules. To avoid background and unintended side reactions, DNA reactants are typically present at low nanomolar concentrations, which means that large-scale DNA circuits may require several hours to days to complete computation. This limitation hinders the practical usability of these systems for their achievable computational tasks.

Recent efforts have been made to accelerate DNA strand displacement circuits, using methods such as surface confinement (*18*, *19*) and strand-displacement DNA synthesis (*20*, *21*). For example, a computing system that uses hybridization chain reactions immobilized on a DNA origami surface has been developed, which can compute two-input dual-rail XNOR logic within an hour (*18*). However, the scalability of surface-based systems is limited because of assembly defects as circuit complexity increases. Similarly, a fast and compact logic circuit was demonstrated using strand-displacing DNA polymerase, which can compute the square-root function in approximately 25 min (*20*), but enzyme stability under fluctuating environmental conditions and mutual interference between DNA oligonucleotides sets a limit on the fidelity of more complex systems.

**Fig. 1.**
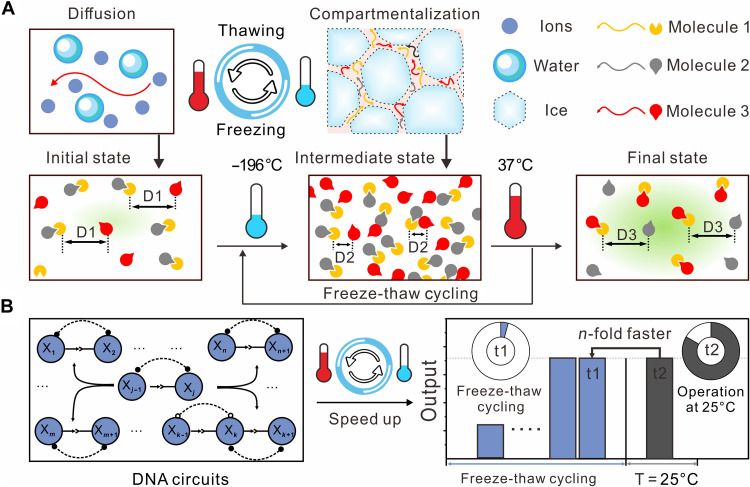
Freeze-thaw cycles driving DNA strand displacement–based computing systems. (**A**) Top row: Freeze-thaw cycling induces diffusion during thawing episodes and compartmentalization during freezing episodes. Bottom row: Molecular complexes experience cycles of diffusive (initial) and compartmentalized (intermediate) states during freeze-thaw cycling, facilitating reactions between active molecules (final state). “D1" to "D3” indicate the intermolecular distances. (**B**) Freeze-thaw cycling for high-speed DNA circuits by subjecting the reaction mixture to repeated eutectic phase freezing, temporarily bringing DNA strands into close physical proximity and enhancing the concentration of active DNA strands. “t1” and “t2” represent the time required for achieving the same output level through repeated freeze-thaw cycling or operation at 25°C.

To address these limitations, we explore an enzyme-free strategy for high-speed DNA circuits without compromising their fidelity through simple freeze-thaw cycles (Fig. 1). This cyclic physicochemical process has previously shown to facilitate ribozyme assembly, prebiotic nucleotide activation, and template-directed RNA copying (*22*–*26*). We show that freeze-thaw cycling can increase the reaction rate of strand displacement by at least 20-fold, even in cases where the reaction is thermodynamically less favored. The iterative temperature and concentration gradients of the cycles continuously enrich active DNA molecules, driving the reaction toward thermodynamic equilibrium. In addition, we find that the topological structure of the eutectic phase of ice can influence the acceleration effect of basic strand displacement reactions by one order of magnitude, providing compartmentalization of DNA-based reactions. The acceleration effect follows the Hofmeister series, where more kosmotropic anions such as SO_4_^2−^ can increase ice crystal growth, resulting in a substantial enhancement of strand displacement reaction rate. Furthermore, we demonstrate that repeated freeze-thaw cycling can markedly increase the reaction rate of molecular digital and neural network computation by up to ~120-fold. A large-scale circuit consisting of ~200 distinct molecules only requires three iterative freeze-thaw cycles to compute results, indicating the broad applicability of this approach for high-speed DNA circuits.

## RESULTS

### Accelerated DNA strand displacement reaction through freeze-thaw cycling

**Fig. 2.**
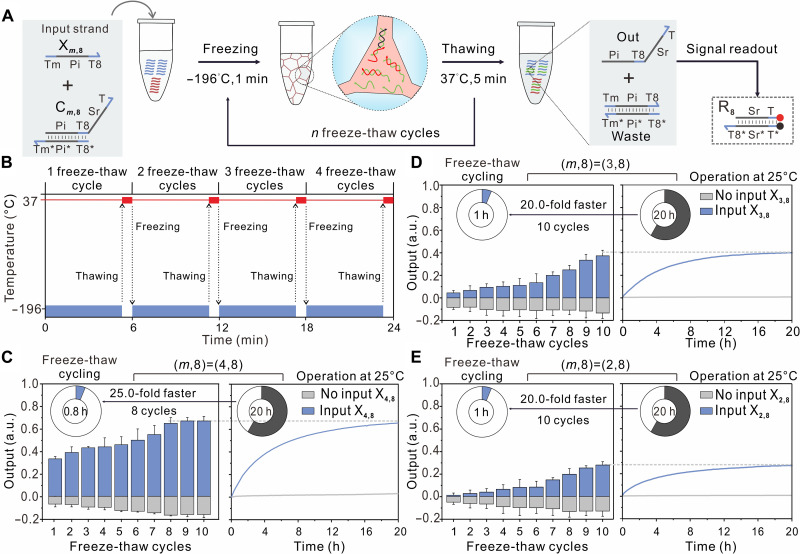
Acceleration of DNA strand displacement reactions through freeze-thaw cycling. (**A**) Binding of an input strand (X_*m*,8_) to a partially double-stranded substrate complex (C_*m*,8_) with the toehold domain Tm* triggers branch migration, resulting in the replacement and release of the output strand (Out). The reporter complex R_8_ stoichiometrically reacts with strand Out, leading to increased fluorescence at 25°C (fig. S1). (**B**) Temperature profile of the freeze-thaw cycling process, where samples undergo freezing at −196°C using liquid N_2_ and subsequent thawing at 37°C. Eutectic conditions occur during both freezing and thawing episodes. (**C** to **E**) Fluorescence response of DNA strand displacement reactions with toeholds of different lengths (4, 3, and 2 nt) under freeze-thaw cycling (left) or operation at 25°C (right), in the presence (blue) or absence (gray) of the input strand (X_*m*,8_). All experiments were conducted with a strand concentration of 50 nM in a tris–ethylenediaminetetraacetic acid (TE) buffer (pH 8.0) containing 12.5 mM MgSO_4_. The numbers within the rings represent the time required for repeated freeze-thaw cycling or operation at 25°C. Data are presented as means ± SD of *n* = 3 independent experiments. a.u., arbitrary units.

We conducted an experimental demonstration to significantly accelerate the simplest nontrivial DNA strand displacement reaction through freeze-thaw cycling (Fig. 2A). Using an iterative regime alternating between freezing (to −196°C) and thawing (to 37°C) throughout the process (Fig. 2B), freezing induced quasi-cellular compartmentalization in the eutectic phase of water ice, bringing single-stranded DNA molecules (X_*m*,8_) and double-stranded complexes (C_*m*,8_) into close physical proximity. This increased the chance of toehold-mediated binding, triggering branch migration reactions. Thawing disrupted both active and unproductive DNA molecules due to the steep drop in DNA and solute concentrations. Consequently, freeze-thaw cycling enabled repeated dehydration and concentration of active DNA molecules, driving the DNA strand displacement reaction toward thermodynamic equilibrium. In our initial experimental demonstration, we selected a strand displacement reaction with a 4–nucleotide (nt) toehold, which typically takes over 20 hours to reach equilibrium at 25°C. However, in the presence of the input strand (X_4,8_), freeze-thaw cycling strongly accelerated the reaction, achieving complete completion within 0.8 hours (8 cycles), increasing the average reaction rate by nearly 25.0-fold compared to the reaction operated at 25°C (Fig. 2C). In addition, in the absence of the input strand (X_4,8_), we observed a 20% drop in fluorescence intensity after eight freeze-thaw cycles, indicating that repeated freeze-thaw cycling could promote fuller hybridization of complementary single strands.

Furthermore, we investigated whether freeze-thaw cycling could accelerate strand displacement reactions with shorter, thermodynamically less favorable toeholds at ambient temperature. After 10 freeze-thaw cycles, we observed a 20.0-fold increase in the average reaction rate for 3- or 2-nt toeholds, with yields increasing as the number of cycles increased (Fig. 2, D and E). These results demonstrate that freeze-thaw cycling can iteratively drive the basic DNA strand displacement reaction toward thermodynamic stabilization. Moreover, thermodynamically less favorable reactions require more cycles of freeze-thaw to reach thermodynamic equilibrium, as shown in table S1.

### The influence of the eutectic ice phase and negative counterions on the acceleration effect of DNA strand displacement reaction

**Fig. 3.**
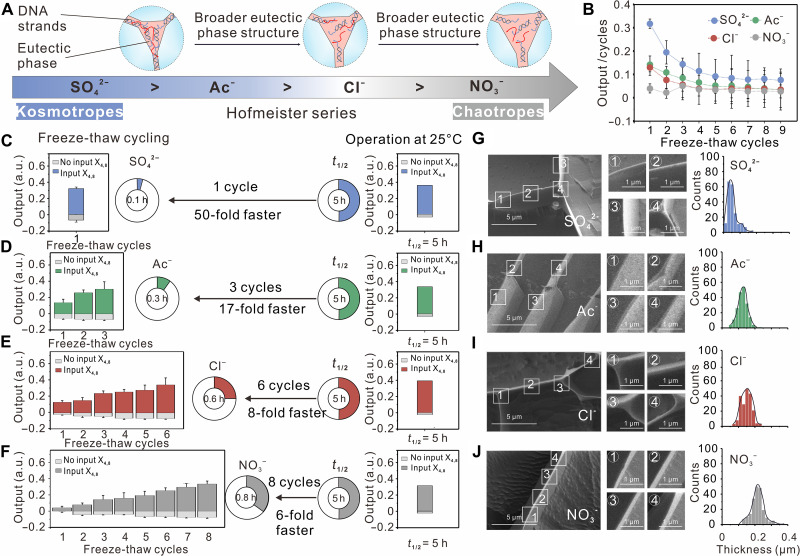
The microstructure of eutectic phase influence on the acceleration effect of DNA strand displacement reactions. (**A**) The influence of negative counterions on the volume of the eutectic phase follows the order of Hofmeister series. (**B**) The average rates of DNA strand displacement with SO_4_^2−^ (blue), Ac^−^ (green), Cl^−^ (red), or NO_3_^−^ (dark gray) are measured by the ratio of the output versus the corresponding cycles of freeze-thaw in the presence of input strand (X_4,8_). (**C** to **F**) Reaction time required to reach the half-completion of the reaction with freeze-thaw cycling or with operation at 25°C in the presence of different counterions [(C) SO_4_^2−^, (D) Ac^−^, (E) Cl^−^, and (F) NO_3_^−^]. The colored and light-gray histograms correspond to reactions in the presence or absence of input strand (X_4,8_), respectively. Data are represented as means ± SD of *n* = 3 independent experiments. (**G** to **J**) Left column: The eutectic phase in ice formed with SO_4_^2−^ (blue), Ac^−^ (green), Cl^−^ (red), or NO_3_^−^ (dark gray), imaged by freeze-fracture SEM. Right column: Histogram representing the thickness of brine vein structures formed with different counterions (bin size = 0.02 μm). Experiments were conducted with all strand concentrations of 50 nM in a TE buffer (pH 8.0) containing 12.5 mM MgSO_4_, (CH_3_COO)_2_Mg, MgCl_2_, or Mg(NO_3_)_2_, respectively.

The microstructure of the eutectic ice phase can be influenced by the identity of negative counterions (*23*), which may affect the concentration effect of active DNA molecules and lead to different acceleration effects of the basic DNA strand displacement reaction through repeated freeze-thaw cycling (Fig. 3A). In our study, we systematically investigated the effect of negative counterions on the rate enhancement of the reaction, specifically altering magnesium counterions (SO_4_^2−^, Ac^−^, Cl^−^, and NO_3_^−^) while maintaining equimolar levels of Mg^2+^ in the solution environment. While minimal changes in reaction rates were observed at 25°C when negative counterions were changed (fig. S2), significant differences were observed through freeze-thaw cycling. As shown in Fig. 3B, the reaction was substantially accelerated after just 1 cycle with any negative counterions, and iterative cycles of freeze-thaw further drove the reaction toward thermodynamic stabilization (fig. S3, A to C). However, we found that the acceleration effects on the reaction were highly ion-dependent when comparing the cycles required to reach half-completion of the reaction, following the sequence of SO_4_^2−^ (1 cycle) < Ac^−^ (3 cycles) < Cl^−^ (6 cycles) < NO_3_^−^ (8 cycles) (Fig. 3, C to F). This ion-dependent behavior aligns with the Hofmeister series, which ranks inorganic salts based on their ability to stabilize proteins in water (*27*, *28*). The introduction of the kosmotropic anion SO_4_^2−^ resulted in a remarkable rate acceleration of the reaction, leading to a ∼50-fold enhancement in reaction speed compared to operation at 25°C. Conversely, replacing SO_4_^2−^ with the more chaotropic anion NO_3_^−^ resulted in only a ∼6-fold acceleration compared to operation at 25°C. In addition, various degrees of decrement in fluorescence intensity were observed in the absence of the input strand (X_4,8_), indicating the ion specificity in promoting hybridization reactions through freeze-thaw cycling (fig. S3D).

Ion specificity has been shown to influence the freezing point, with kosmotropic MgSO_4_ leading to a higher freezing point (*29*), resulting in more extensive freezing and potentially affecting the microstructure of the eutectic phase. Scanning electron microscopy (SEM) imaging revealed lattice structures consisting of brine-filled spaces and ice crystals, which can provide compartmentalization. Quantitative analysis further demonstrated that the thickness of the eutectic ice phase is highly dependent on the anion, following the order in accordance with the Hofmeister series [SO_4_^2−^ (~0.04 μm) < Ac^−^ (~0.14 μm) < Cl^−^ (~0.15 μm) < NO_3_^−^ (~0.22 μm); Fig. 3, G to J]. Comparatively narrower brine vein structures were observed in MgSO_4_ ices compared to alternative counterions, resulting in a more constricted ice microstructure with an increasingly fragmented network of brine veins. This facilitated the increased concentration effect of DNA molecules and the acceleration effect of DNA strand displacement. On the basis of these findings, we can reasonably speculate that the significant rate enhancements achieved through freeze-thaw cycling are attributed to the reduction in eutectic phase volume and the enhanced quasi-cellular compartmentalization, which further supports the observation of SO_4_^2−^ providing optimal speed-up.

### Accelerating cascaded circuits through freeze-thaw cycling

**Fig. 4.**
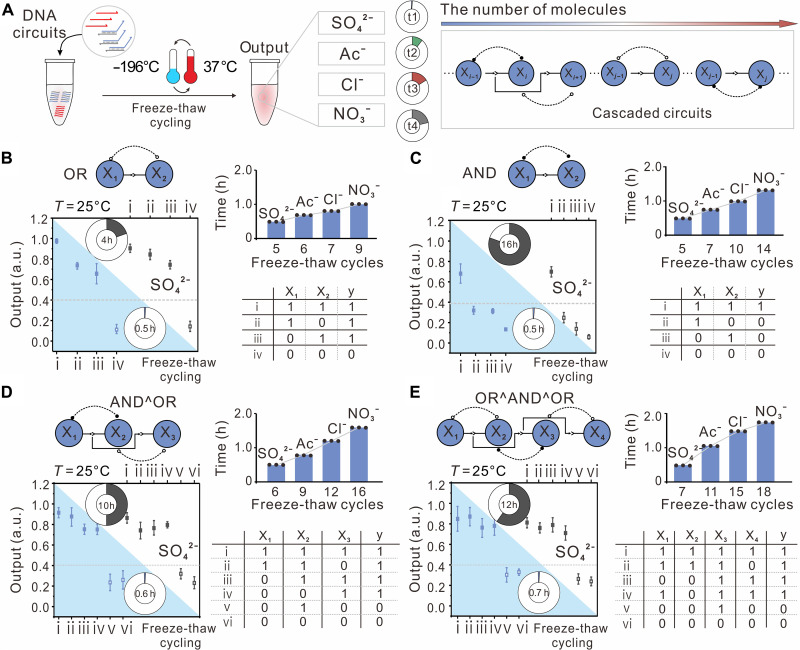
Freeze-thaw cycling accelerates DNA cascaded circuits. (**A**) Schematic illustration of the operations of DNA circuits in the presence of different counterions in freeze-thaw cycling. (**B** to **E**) OR (B) and AND logic gates (C), two-layer cascades AND-OR (D), and three-layer cascades OR-AND-OR (E). Left: The fluorescence levels of the circuit that reached completion with freeze-thaw cycling (blue zone) or with operation at 25°C (blank zone) in the presence of SO_4_^2−^. Right: Freeze-thaw cycles required for circuits to complete computation with different counterions and corresponding truth table for each circuit. Data are represented as means ± SD of *n* = 3 independent experiments. Experiments were conducted in a TE buffer (pH 8.0) containing 12.5 mM MgSO_4_, (CH_3_COO)_2_Mg, MgCl_2_, or Mg(NO_3_)_2_, respectively. The numbers in the rings represent the computation time for circuit with repeated freeze-thaw cycling or operation at 25°C. The gray dashed line marks the threshold value of 0.4 (solid dots for ON and empty dots for OFF).

Having demonstrated that freeze-thaw cycling was able to accelerate basic DNA strand displacement reactions, we explored whether freeze-thaw cycling could also be used to speed up DNA strand displacement circuits of different scales (Fig. 4A). We first designed an elementary logic gate consisted of seven DNA molecules excluding input strands, which can perform either OR or AND by varying the threshold. The circuit contains up to four basic reactions (fig. S4), including switching, transmitting, thresholding, and reporting. The weight molecule *W_i_* can be activated by spontaneous intramolecular conformational switch after hybridization with the switching molecule *Sw_i_*. This allows for precise switching of corresponding circuit components, which then respond to the input signal X*_i_* through a DNA strand displacement reaction, leading to release of the intermediate species. A transmit molecule *Sd_i_* calculates the sum of all intermediate species to transmit the signal from upstream to downstream. A threshold molecule *Th_i_* preferentially absorbs the impinging signal due to a longer toehold, and the signals exceeding threshold can be read out by a reporter molecule with a fluorophore/quencher pair. We used anion SO_4_^2−^ as magnesium counterion for experimental demonstration. When the initial concentration of threshold complex was set to 0 nM, the circuit performed OR logic function, in which the gate computed the output by a completion time of ~4 hours at 25°C (fig. S5A). Specifically, any input species X*_i_* can convert an activate weight molecule to an intermediate product, and then signals can be transmitted downstream and lastly read out by reporter. We then used freeze-thaw cycling to speed up this circuit, and we observed that the output went to clear ON or OFF state for all representative input combinations after only 1 cycle (0.1 hours) (fig. S5B). This speed increase was almost 15-fold compared to operation at 25°C. Furthermore, we found that interactive freeze-thaw cycling would progressively drive the circuit to completion, with circuit yields monotonically increasing with each additional cycle (fig. S5C). Not that 5 cycles of freeze-thaw (0.5 hours) can quickly drive this circuit to thermodynamic equilibrium, corresponding to eightfold speed increase compared with circuit operated at 25°C (Fig. 4B and fig. S5D). When changed the initial concentration of threshold complex from 0 to 40 nM, the circuit transformed to perform AND logic function, in which activated weight molecules can only be converted to an intermediate product with the addition of both inputs, allowing the transmission of intermediate signals to downstream and lastly resulting in fluorescence response once these signals exceed the threshold. Our AND gate computed the output by a completion time of ~16 hours at 25°C (fig. S6A), while the computation time decreased to 0.5 hours through repeated freeze-thaw cycles (Fig. 4C). With all possible inputs from 00 to 11, we observed that only 2 cycles of freeze-thaw can drive this circuit to yield correct computation results, which enhanced the computation speed ∼30-fold compared to operation at 25°C (fig. S6B). With five repeated freeze-thaw cycles, this circuit would be progressively driven to thermodynamic equilibrium (fig. S6, C and D). Notably, 5 cycles of freeze-thaw can rapidly and progressively drive both OR and AND gates to completion within only 0.5 hours, indicating that the acceleration effect of freeze-thaw cycling is similar for circuit of the same size. Note that more cycles are needed for AND gate to correctly perform the logic function than the OR gate due to the higher initial concentration of the threshold complex. As a result, it takes longer for the upstream strand to exceed the threshold for AND gate. In addition, we observed that leak reactions slightly increased in this circuit, but the outputs of circuit still clearly distinguished between OFF and ON states (figs. S5 and S6). Specifically, the level of leak signal after five freeze-thaw cycles is at most three times that of the signal generated after operating the circuit at 25°C for 4 hours. Furthermore, we investigated the negative counterion effect on the acceleration of OR and AND gates (Fig. 4, B and C). By replacing SO_4_^2−^ with other alternative counterions, we found that the acceleration effect of counterions on these basic logic gates through freeze-thaw cycling followed Hofmeister series in comparison with the number of freeze-thaw cycles required to reach completion of this circuit, with the sequence of the SO_4_^2−^ < Ac^−^ < Cl^−^ < NO_3_^−^ (figs. S7 and S8). For example, when replacing kosmotropic anion SO_4_^2−^ with the more chaotropic anion NO_3_^−^, we found that OR logic required four more cycles (AND logic required nine more cycles) of freeze-thaw to reach completion. These observations suggest that iterative freeze-thaw cycling can progressively drive the circuit to run rapidly and correctly. The acceleration effect with the kosmotropic anion SO_4_^2−^ is such that the circuit performs at roughly the same average rate to reach equilibrium even with introduction of threshold complex.

We further investigated whether freeze-thaw cycling could accelerate a two-layer AND-OR circuit consisted of 13 DNA molecules by cascading an AND gate and an OR gate, which takes over 10 hours to complete computation at 25°C (fig. S9, A and B). After 6 cycles of freeze-thaw (0.6 hours), the circuit complete computation for all representative input combinations when using anion SO_4_^2−^ as magnesium counterion, corresponding to a 17-fold increase in speed compared with circuits operated at 25°C (Fig. 4D and fig. S9C). However, when we replaced SO_4_^2−^ with other alternative counterions, the computation speed was noticeably slower, and negative counterion effected the circuit computation in the order of Ac^−^ (9 cycles) < Cl^−^ (12 cycles) < NO_3_^−^ (16 cycles), ranking in the Hofmeister series (fig. S9, D and E). As shown above, when using with the kosmotropic anion replacement SO_4_^2−^ with chaotropic anion such as NO_3_^−^, 10 more cycles are needed for the circuit to get correct function (fig. S9E), which demonstrated that kosmotropic anion has pronounced effects on accelerating the circuit. Moreover, we constructed three-layer cascade OR-AND-OR consisted of 19 DNA molecules, which takes over 12 hours to complete computation at 25°C (fig. S10, A and B). This circuit computation was significantly accelerated through freeze-thaw cycling. Only 7 cycles of freeze-thaw (0.7 hours) were required to complete computation when using kosmotropic anion SO_4_^2−^ as magnesium counterion, corresponding to a 17-fold enhancement in speed compared with circuits operated at 25°C (Fig. 4E and fig. S10C). When we replaced SO_4_^2−^ with other alternative counterions, we found that the acceleration effect of counterions also follows the Hofmeister series [SO_4_^2−^ (7 cycles) < Ac^−^ (11 cycles) < Cl^−^ (15 cycles) < NO_3_^−^ (18 cycles); fig. S10, C to E]. In general, the number of freeze-thaw cycles for circuits to complete computation increases with the circuit size. For instance, 5 cycles for a single-layer circuit, 6 cycles for a two-layer circuit (AND-OR), and 7 cycles for a three-layer circuit (OR-AND-OR) with SO_4_^2−^ as magnesium counterion. Moreover, kosmotropic anion SO_4_^2−^ shows great superiority to accelerate circuit with different scales through freeze-thaw cycling, and the consistency of the property illustrates the effective concentration effect of SO_4_^2−^ counterion during freeze-thaw cycling.

### Accelerating large-scale DNA neural network circuit through freeze-thaw cycling

**Fig. 5.**
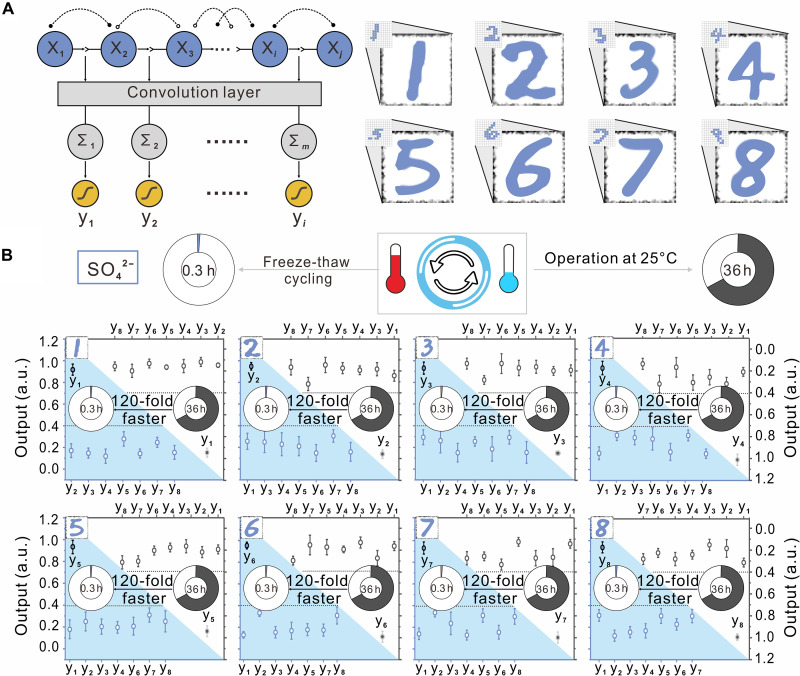
Accelerating large-scale DNA neural network circuit through freeze-thaw cycling. (**A**) Left: Circuit diagram of the DNA-based ConvNet for pattern recognition, with each output y*_i_* corresponding uniquely to specific handwritten digits. Right: Eight target patterns representing digits ‘1’ to ‘8’, displayed in a 12 × 12 grid format. Each blue pixel represents a 1, and each white pixel represents a 0, indicating the presence or absence of a specific input strand (see fig. S11). (**B**) Fluorescence levels used to characterize the recognition behavior, comparing freeze-thaw cycling (blue zone, left *y* axis) and operation at 25°C (blank zone, right *y* axis). Data collected from three repeated experiments per digit. Solid dots represent target patterns, while empty dots represent out-of-class target patterns. Data are presented as means ± SD of *n* = 3 independent experiments. Experiments were conducted in a TE buffer (pH 8.0) containing 12.5 mM MgSO_4_. The numbers in the rings indicate the time required to complete the pattern recognition with repeated freeze-thaw cycling or operation at 25°C. The dotted light-gray line represents the threshold value of 0.4 (solid dots for ON and empty dots for OFF).

To demonstrate the broad applicability of freeze-thaw cycling, we conducted experimental tests to accelerate large-scale circuits comprising hundreds of DNA molecules. Building upon our previous work (*17*), we constructed a DNA-based ConvNet capable of recognizing and remembering eight handwritten digits (Fig. 5A). Initially, we developed an optimized model by training and validating it using the MNIST database, achieving a 90% accuracy. This model consisted of input features, associated convolution kernels, and convolution operations performed on these inputs. Subsequently, we translated all model parameters and mathematical functions into a class of DNA molecules that react in a predetermined order, enabling the construction of a DNA ConvNet circuit at the molecular level (fig. S11). To experimentally verify the performance of the DNA circuit, we used eight test patterns. However, we observed that at an operating temperature of 25°C, the circuit required approximately 36 hours to produce the desired outputs, limiting the practical application of DNA neural networks in biological contexts (Fig. 5B). By using repeated freeze-thaw cycling, we found that the computation speed of the network circuit was significantly accelerated. With 3 cycles of freeze-thaw and the use of SO_4_^2−^ as the magnesium counterion, each input pattern triggered the desired outputs in less than 20 min, resulting in a ∼120-fold increase in computation speed (Fig. 5B and fig. S12). These results highlight that freeze-thaw cycling can facilitate rapid and accurate computation in large-scale circuits for complex tasks. Furthermore, we found that the acceleration effect of negative counterions on the speed of the DNA network followed the Hofmeister series during freeze-thaw cycling, with the sequence being SO_4_^2−^ (3 cycles) < Ac^−^ (4 cycles) < Cl^−^ (5 cycles) < NO_3_^−^ (7 cycles) (figs. S12 to S15). This remarkable acceleration effect may arise from the synchronous enhancement of massively parallel operations through freeze-thaw cycling, which accelerates each DNA strand displacement reaction, leading to an overall increase in circuit computation speed. These findings indicate that freeze-thaw cycling exhibits a more substantial acceleration effect for circuits that operate independently and in parallel.

## DISCUSSION

In this study, we have introduced freeze-thaw cycling as a straightforward and versatile method to achieve high-speed computation in DNA strand displacement circuits, reducing computation times from hours to minutes. The formation of a highly structured eutectic ice phase during freeze-thaw cycling facilitates quasi-cellular compartmentalization, which likely leads to the enrichment of active DNA molecules and accelerates computation speed. We have demonstrated that freeze-thaw cycling can significantly promote DNA strand displacement reactions that are thermodynamically less favored, increasing the reaction rate by at least 20-fold. Furthermore, we have systematically investigated the differential acceleration effects of negative counterions on the reactions during freeze-thaw cycling. We have observed that the acceleration effect follows the Hofmeister series, with more kosmotropic anions (such as SO_4_^2−^) substantially enhancing the speed of DNA strand displacement compared to chaotropic anions (such as NO_3_^−^), primarily due to the narrower brine vein structure. Moreover, we have demonstrated the broad applicability of this approach by accelerating DNA circuits of various scales, including logic gates, cascade circuits, and large-scale neural network circuits. Through iterative freeze-thaw cycling, the large-scale neural network circuit composed of hundreds of DNA strands can achieve rapid computation in less than 20 min, with a ∼120-fold increase in computation speed (table S2). This indicates the enormous potential of this method for parallelism acceleration in molecular operations.

Our method stands out because of its simplicity and wide applicability. Freeze-thaw cycling can accelerate DNA strand displacement circuits without the need for additional complex components, such as enzymes for operation or DNA origami scaffolds for circuit design. The only requirement is repeated freeze-thaw cycles to achieve fast and accurate circuit operation. This method can potentially be scaled up to accelerate other artificially designed DNA molecular systems, such as winner-take-all DNA neural networks. With its simplicity and general applicability, we anticipate that freeze-thaw cycling will facilitate the development of more powerful large-scale DNA computational systems. In addition, DNA circuits can be adapted to process a broader range of signal classification tasks, including diagnostic applications for detecting complex disease profiles involving microRNA signals. This suggests that freeze-thaw cycling holds promise for fast sensing applications that enable faster diagnoses or prognoses.

## MATERIALS AND METHODS

### DNA sequences and code availability

All DNA strands have long recognition domains and short toehold domains, which are functionally independent. Thus, we design the sequence at the domain level. To avoid unwanted secondary structures and undesired strand interactions, we used a three-letter code (A, T, and C) for sequence design to prevent the co-occurrence of G and C in the same strand. Domain sequences include runs of less than four consecutive A’s or T’s or less than three consecutive C’s to reduce synthesis errors. All domain sequences were kept with 30 to 70% C content. First, we randomly generated and screened a library of sequences. To validate the candidate sequences, we used NUPACK (*30*) to check the binding energy and specificity. Last, these sequences were assigned to the circuits for further experiments. All DNA sequences are given in table S3. The ConvNet model for eight-pattern recognition was used in our work, which is available on the Code Ocean (*17*) (https://codeocean.com/capsule/0704369/tree/v1).

### DNA oligonucleotide synthesis

All DNA strands were purchased from Sangon Biotech, in which the unlabeled DNA strands were purified using ultra–polyacrylamide gel electrophoresis and the labelled DNA strands were purified using high-performance liquid chromatography. All lyophilized strands were resuspended at 100 μM in tris–ethylenediaminetetraacetic acid (TE) buffer with 12.5 mM Mg^2+^, pH 8.0, and stored at 4°C.

### Annealing protocol and buffer condition

All complexes were annealed at 20 μM. Reporter complexes were annealed with a 20% excess of bottom quencher strands. While the other complexes were annealed with a 1:1 ratio of top strands and bottom strands, and the weight complexes (*W_i_* and *N*_*Wt*,*Ii*,*j*_) were annealed with top and bottom strands in a 1:1:1 ratio. The buffer for experiments was 1× TE with 12.5 mM MgSO_4_ [or if indicated, (CH_3_COO)_2_Mg, MgCl_2_, and Mg(NO_3_)_2_] at pH 8.0. The reaction mix was then annealed in a thermal cycler (Life Technologies) by heating to 95°C for 5 min and slowly cooling to 20°C at a rate of 0.1°C/8 s and then held at 4°C. The hybridized molecules were stored at 4°C for further use.

### Protocol for the freeze-thaw cycling

The temperature profile of freeze-thaw cycling was generated using the following steps: (i) transferring the tube to a liquid nitrogen environment at −196°C for 1 min, (ii) setting the water bath to a target temperature of 37°C and maintaining it for 5 min, and (iii) returning to step (i).

### Fluorescence spectroscopy

A spectrofluorimeter (Fluorolog-max, Horiba) was used for fluorescence experiments. Four kinds of fluorophore were used: 6-Carboxyfluorescein (FAM: excitation, 492 nm; emission, 518 nm), Carboxy-X-rhodamine (ROX: excitation, 585 nm; emission, 605 nm), Hexachlorofluorescein (HEX: excitation, 535 nm; emission, 556 nm), and Cyanine 5.5 (Cy5.5: excitation, 685 nm; emission, 705 nm). Previous studies have demonstrated no interference between these fluorophores (*17*). In the eight-pattern recognition, eight fluorescence outputs were recorded using four distinct fluorophores (*17*). Each experiment was conducted twice, with half of the outputs associated with fluorophore-labeled reporters and the remaining half associated with nonfluorophore-labeled reporters. All eight outputs can be observed simultaneously by combining their trajectories in a single plot. For the rest of the circuits, only FAM was used. In freeze-thaw cycling process, reporter complex R_8_ with a toehold of 8 nt was added after last freeze-thaw cycle to quickly react with output strands to yield an increased fluorescence signal. Note that using a separate reporter complex in freeze-thaw cycling rather than directly labeling complexes or input strands with fluorophores and quenchers would prevent the potential impact of repeated freeze-thaw cycles on fluorophores. Here, the indirect reporter complex R_8_ isolates this effect from characterizing the acceleration efficiency.

### Fluorescence data normalization

All data were normalized from raw fluorescence levels to relative concentrations of output signals. Each set of parallel experiments was conducted for the same circuit with various inputs, and the outputs were normalized together for data analysis. For reactions with freeze-thaw cycling, reporter complex R_8_ was used to read the output. All the circuit strands except the input strands and reporter complex R_8_ (toehold = 8 nt) were mixed. The half of the well-mixed solution was allowed to react for the same length of time that was required to reach initial steady state when reactions were operated at 25°C, and then reporter complex R_8_ (toehold = 8 nt) was added. The minimum level was recorded as the baseline (output = 0). The other half of the well-mixed solution was added with input strands and allowed to react for the same length of time that was required to reach completion when reactions were operated at 25°C, and then reporter complex R_8_ (toehold = 8 nt) was added. The maximum level (output = 1) was the maximum of all data points. For reactions operated at 25°C, the fluorophore was directly labeled in complex C_*m*,8,R_ to read the output. All strands except the input strands are mixed, and the initial steady-state signal was recorded as the baseline (output = 0). Then, the experiment was paused for adding the input strands and subsequent mixing by a pipette. For the same fluorophore in parallel experiments, where at least one of the output signals would reach a plateau in the end, the maximum level (output = 1) was obtained from the mean of the last five data points. While for experiments where none of output signals reached completion, we conducted a postexperiment triggering step by introducing an excess of inputs to the gates responsible for directly generating the circuit outputs. The maximum level (output = 1) was determined by calculating the average of the last five data points after the output signals reached a plateau.

### Scanning electron microscopy

Samples were prepared for cryo-SEM in the presence of specific counterion buffers by loading a protruding drop of solution into the ends of copper tubes (1 mm in diameter) and plunging into liquid N_2_. Frozen specimen was moved to a preparation chamber (Alto 2500) where they were freeze-fractured with a cold scalpel blade. The temperature of the chamber was raised (−90°C for 10 min) to allow sublimation of water from ice crystals, resulting in fragmented network of brine veins. The specimen was imaged using cryo-SEM (Hitachi S-4800) at −160°C.

### Statistical analysis

Data are presented as means ± SD of *n* = 3 independent experiments.
