# Supplementary material for: Accelerating DNA computing via freeze-thaw cycling
Source: Sci Adv. 2023 Aug 25;9(34):eaax7983. doi: 10.1126/sciadv.aax7983 (PMC10456841; doi:10.1126/sciadv.aax7983)
Supplement: Supplementary file 1 — Figs. S1 to S15 Tables S1 and S2 Legend for table S3 [file sciadv.aax7983_sm.pdf]

Science Advances
AAAS

Supplementary Materials for

Accelerating DNA computing via freeze-thaw cycling

Yun Zhu *et al.*

Corresponding author: Hao Pei, peihao@chem.ecnu.edu.cn

Sci. Adv. **9**, eaax7983 (2023)
DOI: 10.1126/sciadv.aax7983

The PDF file includes:

Figs. S1 to S15
Tables S1 and S2
Legend for table S3

Other Supplementary Material for this manuscript includes the following:

Table S3

Figures and Tables

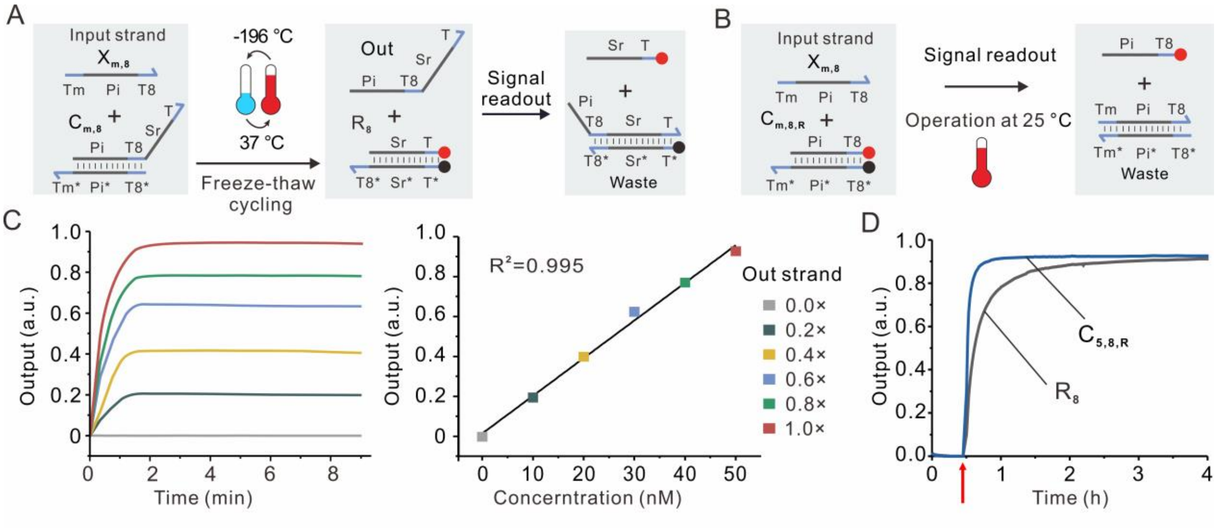

Fig. S1. Schematic of the experimental system used for reporting when operated in freeze-thaw cycling or operated at 25 °C. The DNA implementation of reporting either with (**A**) freeze-thaw cycling or with (**B**) operation at 25 °C. In freeze-thaw cycling, reporter R_8 (toehold = 8 nt) reacted with an output strand (Out) released from $C_{m,8}$, converting the output signal (Out) to a fluorescence signal. When operation at 25 °C, reporter $C_{m,8,R}$ was used to directly reacted with an input strand ($X_{m,8}$), yielding a fluorescence signal. The red dot represents the fluorescent group of FAM, and the black dot represents the quencher group of BHQ-1. (**C**) Left: The fluorescence reporting kinetics (left) of different concentrations of strand Out reacting with R_8 in room temperature; Right: Linear relationships were obtained between the concentrations of strand Out and the steady-state fluorescence response. The data showed that reporting could be implemented rapidly within 5 min and reporter R_8 could react stoichiometrically with product generated after freeze-thaw cycling. Reporter R_8 was added in solution with relative concentrations of 1×. (**D**) Gray curve: R_8 were mixed in solution with relative concentrations of 1×, input strand ($X_{5,8}$) and complex strand ($C_{5,8}$) were then added with relative concentrations of 1×; Blue curve: Reporter ($C_{5,8,R}$) was added in solution with relative concentrations of 1×, input strand ($X_{5,8}$) was then added at 1×. We observed that two reactions achieved the same equilibrium yields, which indicates that the separate reporter R_8 could accurately report the output signal generated in freeze-thaw cycling process. The standard concentration is 50 nM (1× = 50 nM).

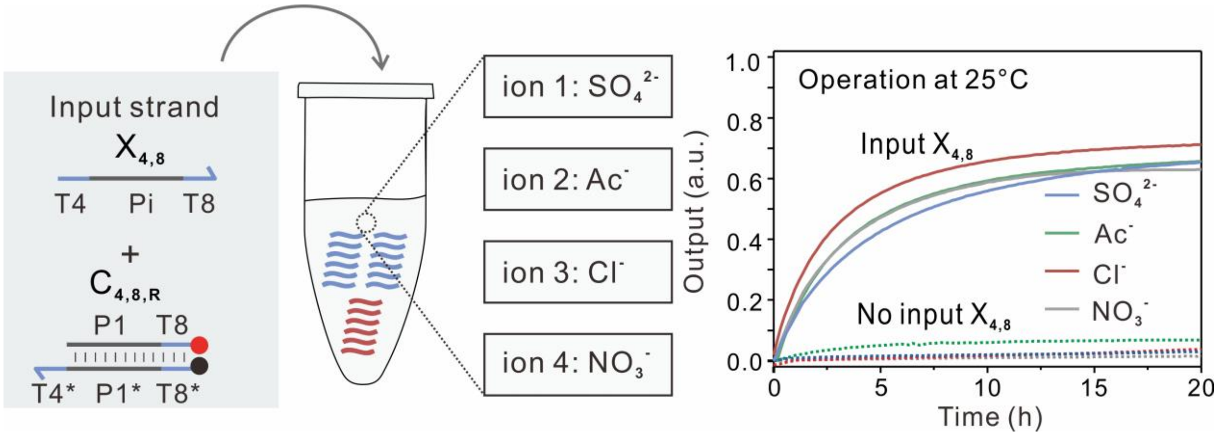

Fig. S2. Kinetic characterization of DNA strand displacement reactions operated at 25 °C, when using SO_4^{2-}, Ac^-, Cl^-, or NO_3^- as magnesium counterion, respectively. The concentrations of $C_{4,8,R}$ was at 1×. Input strand ($X_{4,8}$) was at 1× (solid lines), or 0× (dotted lines). Experiments were conducted in a TE buffer (pH 8.0) containing 12.5 mM $MgSO_4$, $(CH_3COO)_2Mg$, $MgCl_2$, or $Mg(NO_3)_2$, respectively. The standard concentration is 50 nM (1× = 50 nM).

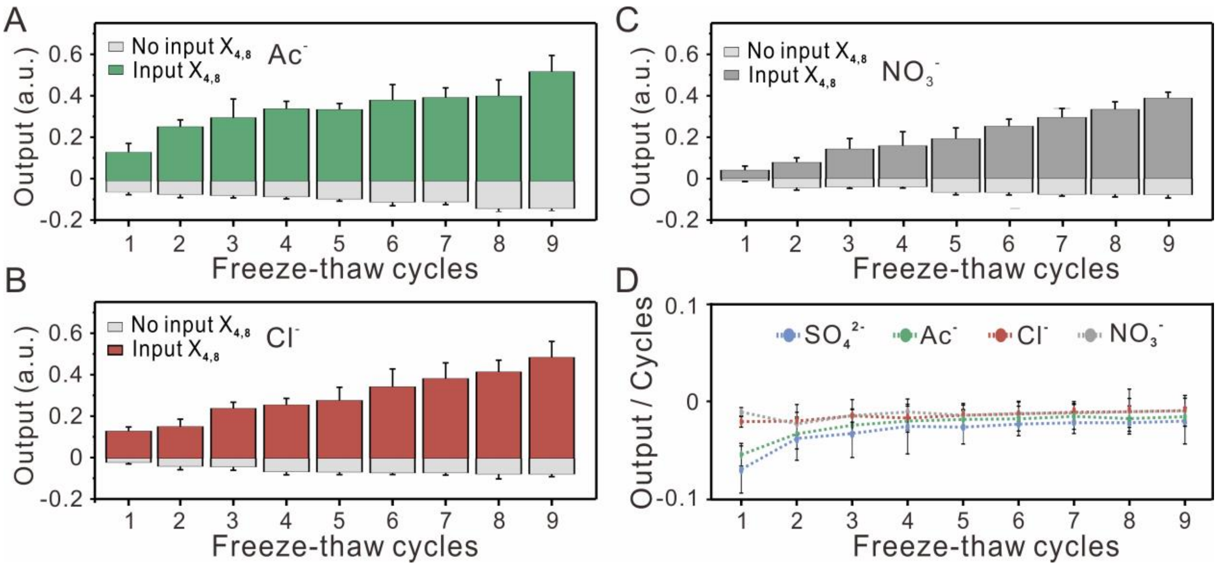

Fig. S3. Freeze-thaw cycling accelerates DNA strand displacement reaction in the presence of different counterions. (**A to C**) Repeated freeze-thaw cycling sped up the DNA strand displacement reaction in the presence of different counterions (**A**: Ac^-, **B**: Cl^-, **C**: NO_3^-). (**D**) The average rates of the DNA hybridization of complementary DNA strands was calculated as the ratio of the corresponding output level versus the required cycles of freeze-thaw in the absence of input strand ($X_{4,8}$). Data are represented as mean ± s.d. of n = 3 independent experiments.

A

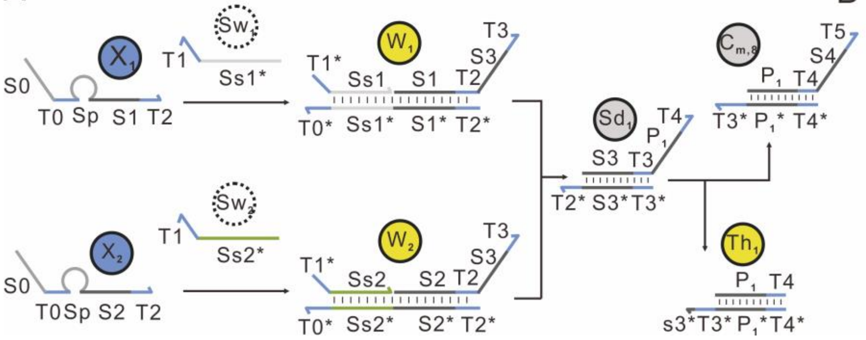

B

	Number of species
Input ,X_i	2
Weight ,W_i	2
Switching,Sw_i	2
Transmit,Sd_i	1
Threshold,Th_i	1
Complex,$C_{m,8}$	1
Reporter,$C_{m,8,R}$	1
Reporter,R_8	1

Fig. S4. Single-layer circuit. (**A**) Domain-level of single-layer DNA circuit. (**B**) Number of distinct species in the circuit. Weight complex (W_i), switching complex (Sw_i), transmit complex (Sd_i), complex ($C_{m,8}$) and reporter ($C_{m,8,R}$ or R_8) were at 1×, 2×, 4×, 1×, and 1×, respectively (standard concentration 1× = 50 nM). Reporter $C_{m,8,R}$ was used for signal readout at 25 °C, and reporter R_8 was used for signal readout during freeze-thaw cycling. Input strands were at 0× or 2×. Circuit computes either OR or AND through the adjustment of initial concentration of the threshold (Th_i).

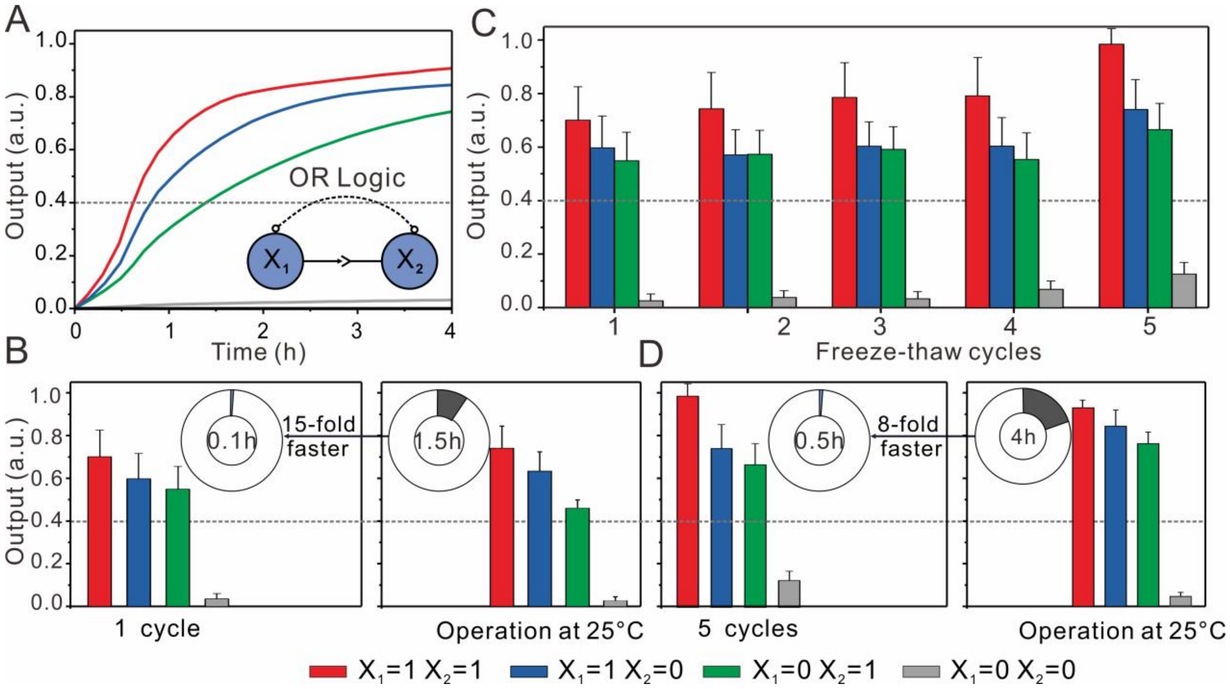

Fig. S5. Freeze-thaw cycling accelerates OR logic gate. (**A**) The fluorescence kinetics data of OR logic gate when operated at 25 ℃. (**B**) Comparisons of the fluorescence level of outputs after 1 freeze-thaw cycle (left) and at 1.5 h operated at 25 ℃ (right). (**C**) The fluorescence level of outputs through repeated freeze-thaw cycles. (**D**) Comparisons of the fluorescence level of outputs after 5 freeze-thaw cycles (left) and at 4 h operated at 25 ℃ (right). Data are represented as mean ± s.d. of n = 3 independent experiments. The initial concentration of threshold (Th_i) was 0×. Experiments were conducted in TE buffer (pH 8.0) containing 12.5 mM $MgSO_4$. The gray dotted line marks the threshold value of 0.4. The standard concentration is 50 nM (1× = 50 nM).

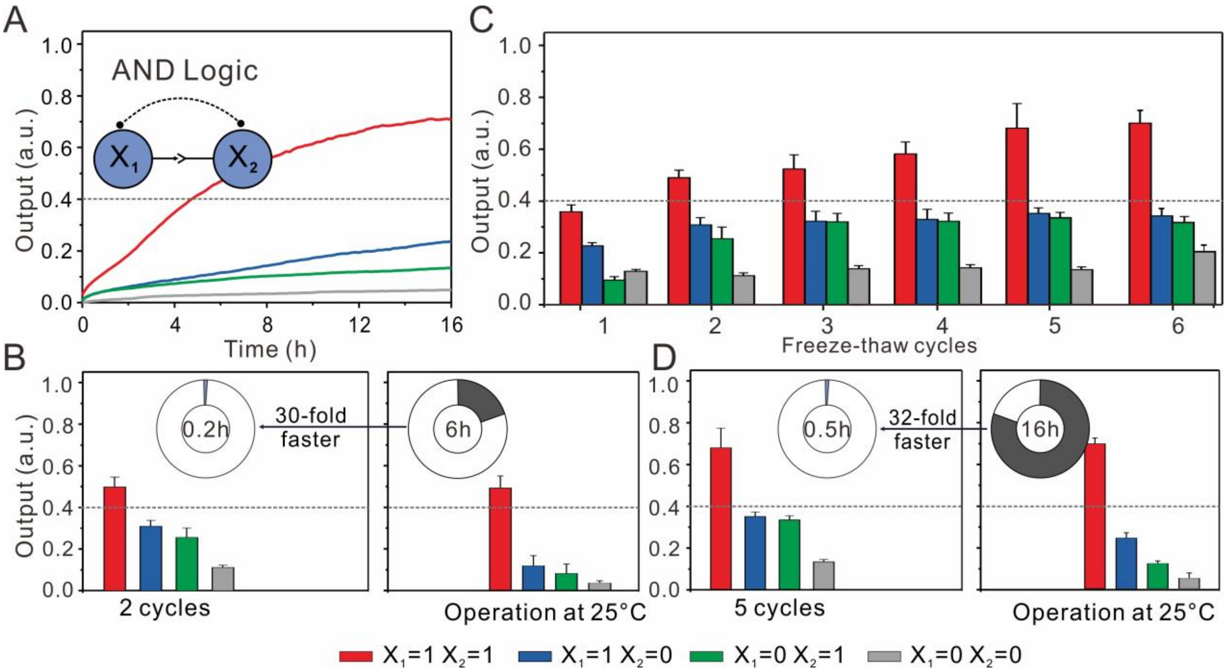

Fig. S6. Freeze-thaw cycling accelerates AND logic gate. (**A**) The fluorescence kinetics data of AND logic gate when operated at 25 °C. (**B**) Comparisons of the fluorescence level of outputs after 2 freeze-thaw cycle (left) and at 6 h operated at 25 °C (right). (**C**) The fluorescence level of outputs through repeated freeze-thaw cycles. (**D**) Comparisons of the fluorescence level of outputs after 5 freeze-thaw cycles (left) and at 16 h operated at 25 °C (right). Data are represented as mean ± s.d. of n = 3 independent experiments. The initial concentration of threshold (Th_i) is 0.8× (Standard concentration 1× = 50 nM). Experiments were conducted in TE buffer (pH 8.0) containing 12.5 mM $MgSO_4$. The gray dotted line marks the threshold value of 0.4.

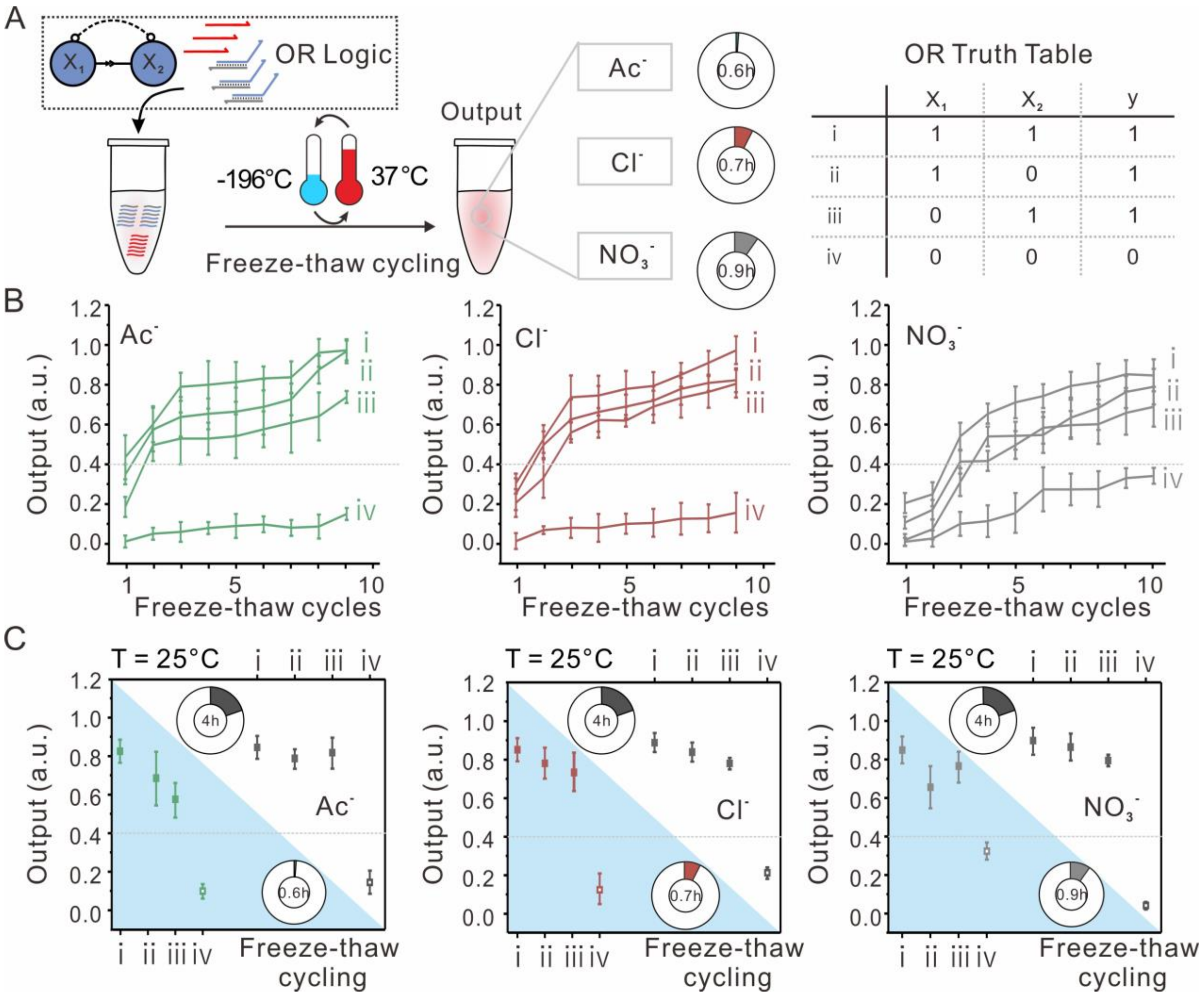

Fig. S7. Ion identity affects the acceleration effect of OR logic gate. (**A**) Left: Freeze-thaw cycling speeds up OR logic in the presence of different counterions; Right: Truth table. (**B**) Fluorescence levels of the OR logic gate through repeated freeze-thaw cycles when using Ac^-(green), Cl^- (red), and NO_3^- (gray) as magnesium counterion, respectively. (**C**) The fluorescence levels of the circuit that reached completion either with freeze-thaw cycling (blue zone) or with operation at 25 °C (blank zone) when using different magnesium counterions (Ac^-, Cl^-, NO_3^-). Data are represented as mean ± s.d. of n = 3 independent experiments. The numbers in the rings represented the time required for the repeated freeze-thaw cycling or operation at 25 °C. Experiments were conducted in a TE buffer (pH 8.0) containing 12.5 mM $(CH_3COO)_2Mg$, $MgCl_2$, or $Mg(NO_3)_2$, respectively. The gray dotted line marks the threshold value of 0.4 (solid dots for ON, empty dots for OFF).

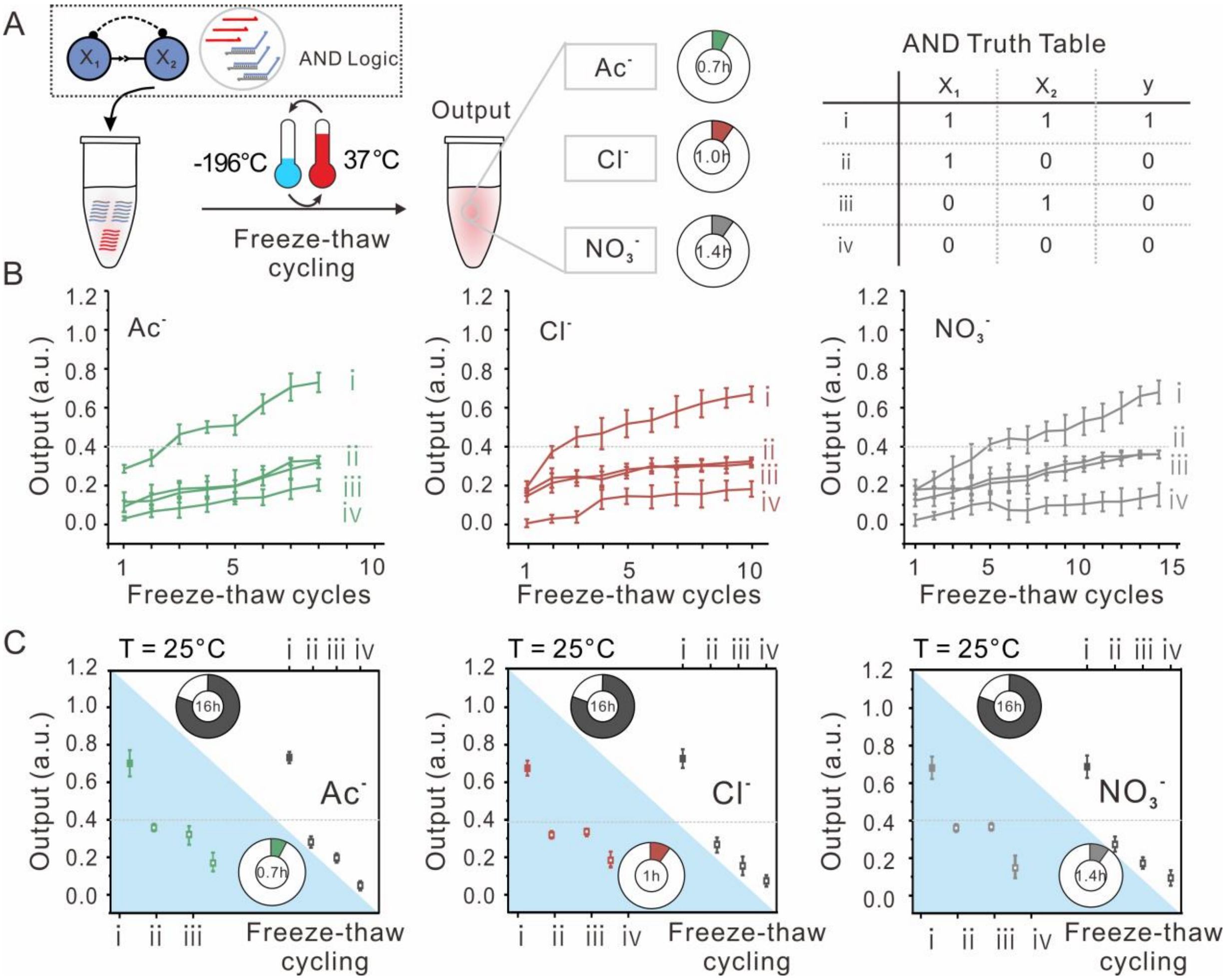

Fig. S8. Ion identity affects the acceleration effect of AND logic gate. (**A**) Left: Freeze-thaw cycling speeds up AND logic in the presence of different counterions; Right: Truth table. (**B**) Fluorescence levels of the AND logic gate through repeated freeze-thaw cycles in the presence of Ac^-(green), Cl^- (red), and NO_3^- (gray), respectively. (**C**) The fluorescence levels of the circuit that reached completion either with freeze-thaw cycling (blue zone) or with operation at 25 °C (blank zone) when using different magnesium counterions (Ac^-, Cl^-, NO_3^-). Data are represented as mean ± s.d. of n = 3 independent experiments. The number in the rings represented the time required for repeated freeze-thaw cycling or operation at 25 °C. Experiments were conducted in a TE buffer (pH 8.0) containing 12.5 mM $(CH_3COO)_2Mg$, $MgCl_2$, or $Mg(NO_3)_2$, respectively. The gray dotted line marks the threshold value of 0.4 (solid dots for ON, empty dots for OFF).

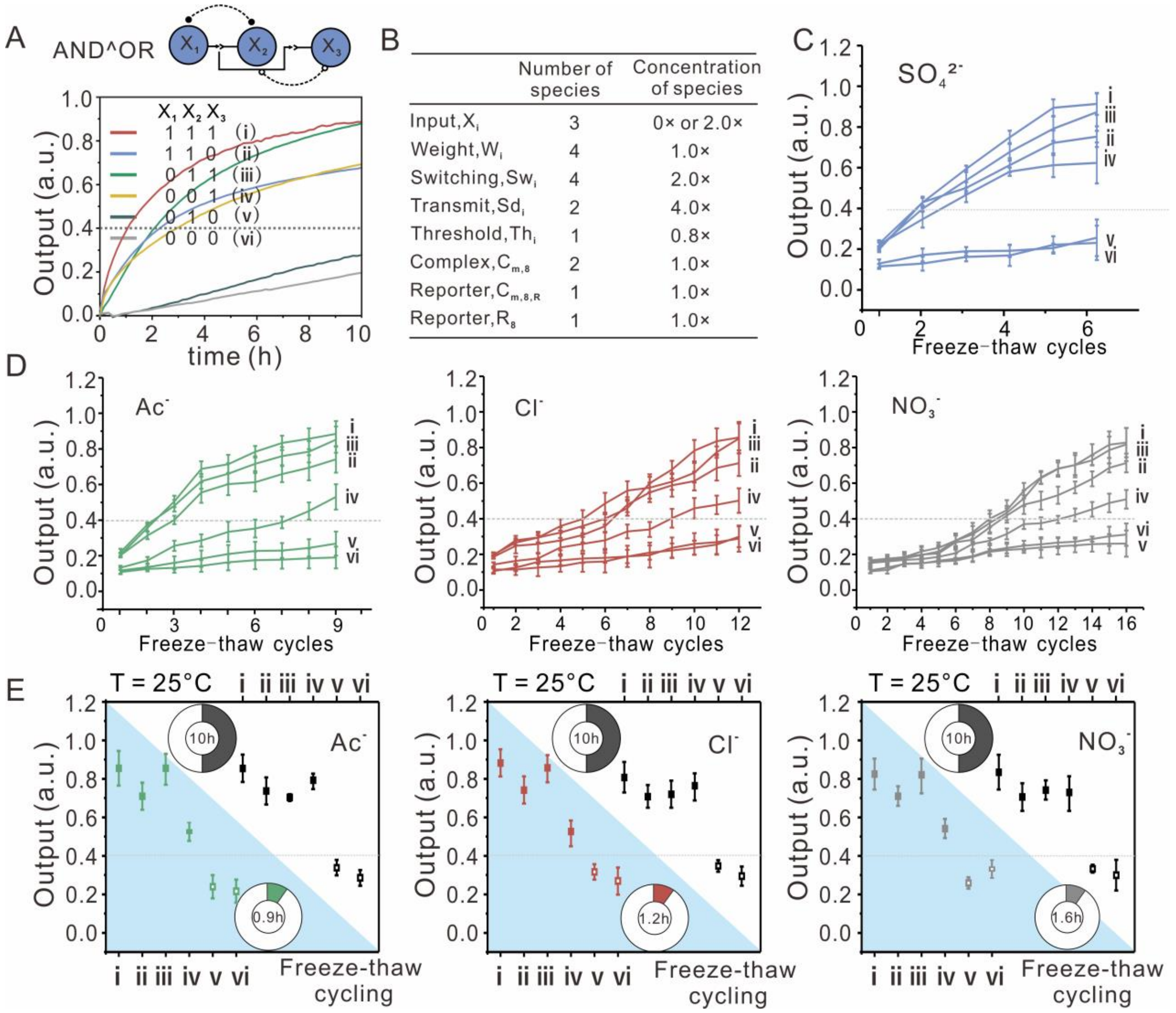

	Number of species	Concentration of species
Input, X_i	3	0× or 2.0×
Weight, W_i	4	1.0×
Switching, Sw_i	4	2.0×
Transmit, Sd_i	2	4.0×
Threshold, Th_i	1	0.8×
Complex, $C_{m,8}$	2	1.0×
Reporter, $C_{m,8,R}$	1	1.0×
Reporter, R_8	1	1.0×

Fig. S9. Two-layer circuit. (**A**) Kinetics experiments. (**B**) Distinct species and corresponding concentrations used in the circuit. Repeated freeze-thaw cycles were used to accelerate two-layer DNA circuit in the presence of (**C**) SO_4^{2-} or other counterions (**D**) Ac^-, Cl^-, NO_3^-. (**E**) The fluorescence levels of the circuit that reached completion either with freeze-thaw cycling (blue zone) or with operation at 25 °C (blank zone) when using different magnesium counterions (Ac^-, Cl^-, NO_3^-). Data are represented as mean ± s.d. of n = 3 independent experiments. Experiments were conducted in a TE buffer (pH 8.0) containing 12.5 mM $MgSO_4$, $(CH_3COO)_2Mg$, $MgCl_2$, or $Mg(NO_3)_2$, respectively. The numbers in the rings represented the time required for repeated freeze-thaw cycling or operation at 25 ℃. The gray dotted line marks the threshold value of 0.4 (solid dots for ON, empty dots for OFF).

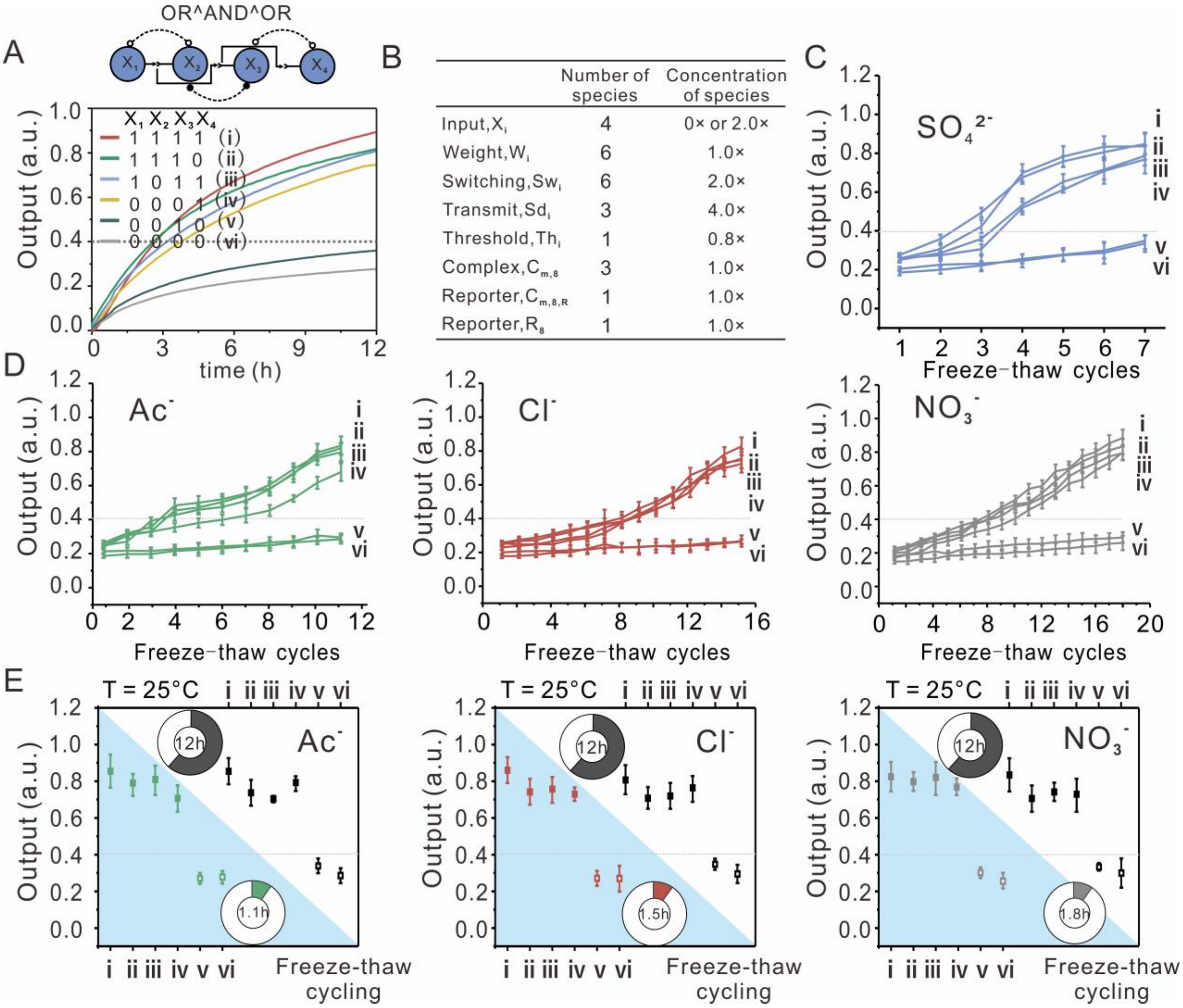

Fig. S10. Three-layer circuit. (**A**) Kinetics experiments. (**B**) Distinct species and corresponding concentrations used in the circuit. Repeated freeze-thaw cycles were used to accelerate three-layer DNA circuit in the presence of (**C**) SO_4^{2-} or other counterions (**D**) Ac^-, Cl^-, NO_3^-. (**E**) The fluorescence levels of the circuit that reached completion either with freeze-thaw cycling (blue zone) or with operation at 25 °C (blank zone) when using different magnesium counterions (Ac^-, Cl^-, NO_3^-). Data are represented as mean ± s.d. of n = 3 independent experiments. Experiments were conducted in a TE buffer (pH 8.0) containing 12.5 mM $MgSO_4$, $(CH_3COO)_2Mg$, $MgCl_2$, or $Mg(NO_3)_2$, respectively. The numbers in the rings represented the time required for repeated freeze-thaw cycling or operation at 25 ℃. The gray dotted line marks the threshold value of 0.4 (solid dots for ON, empty dots for OFF).

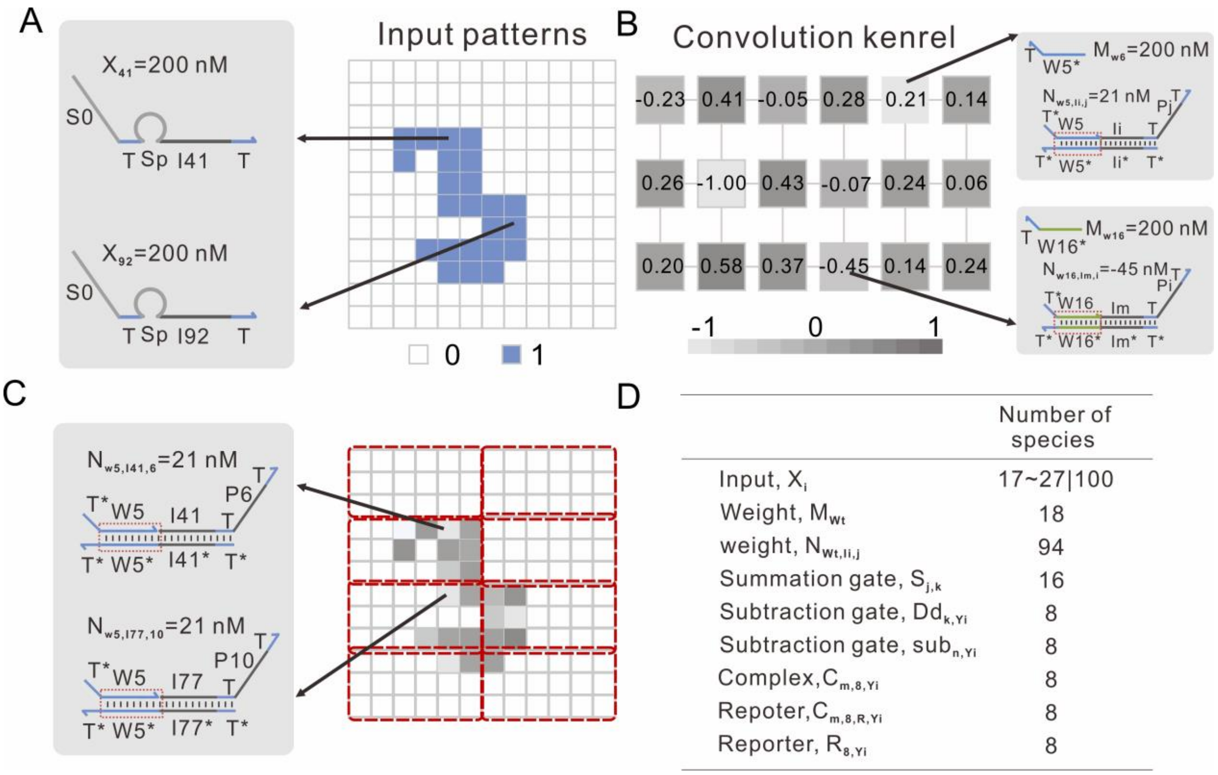

	Number of species
Input, X_i	17~27|100
Weight, M_{wt}	18
weight, $N_{wt,Ii,j}$	94
Summation gate, $S_{j,k}$	16
Subtraction gate, $Dd_{k,Yi}$	8
Subtraction gate, $sub_{n,Yi}$	8
Complex, $C_{m,8,Yi}$	8
Repoter, $C_{m,8,R,Yi}$	8
Reporter, $R_{8,Yi}$	8

Fig. S11. The DNA implementation of DNA-based convolutional neural network (ConvNet). (**A**) The original handwritten symbols were converted to binary patterns where each 1 or 0 corresponds to the presence and absence of the input strand. The concentration of each input strand is 200 nM. (**B**) The DNA implementation of the convolution kernel with dimensions of 3×6. Each weight value of each pixel in the convolution kernel determines the concentration of the weight substrate molecule $N_{Wt,Ii,j}$ (for example, 21 nM for the 5th pixel). Each weight can be encoded in a distinct sequence of weight tuning domain (for example, blue domains W5* and green domains W16*). Note that positive and negative weights are implemented by using different output sequences of $N_{Wt,Ii,j}$ (for example, Pj in $N_{W5,Ii,j}$ and Pi in $N_{W16,Im,i}$). (**C**) Each receptive region (red dashed box) of a '3' reacts with the same convolution kernel to export feature maps, where weight tuning molecule M_{Wt} activates the corresponding weight substrate molecule $N_{Wt,Ii,j}$ in eight receptive regions in parallel. (**D**) The number of distinct molecular species used in the DNA-based ConvNet.

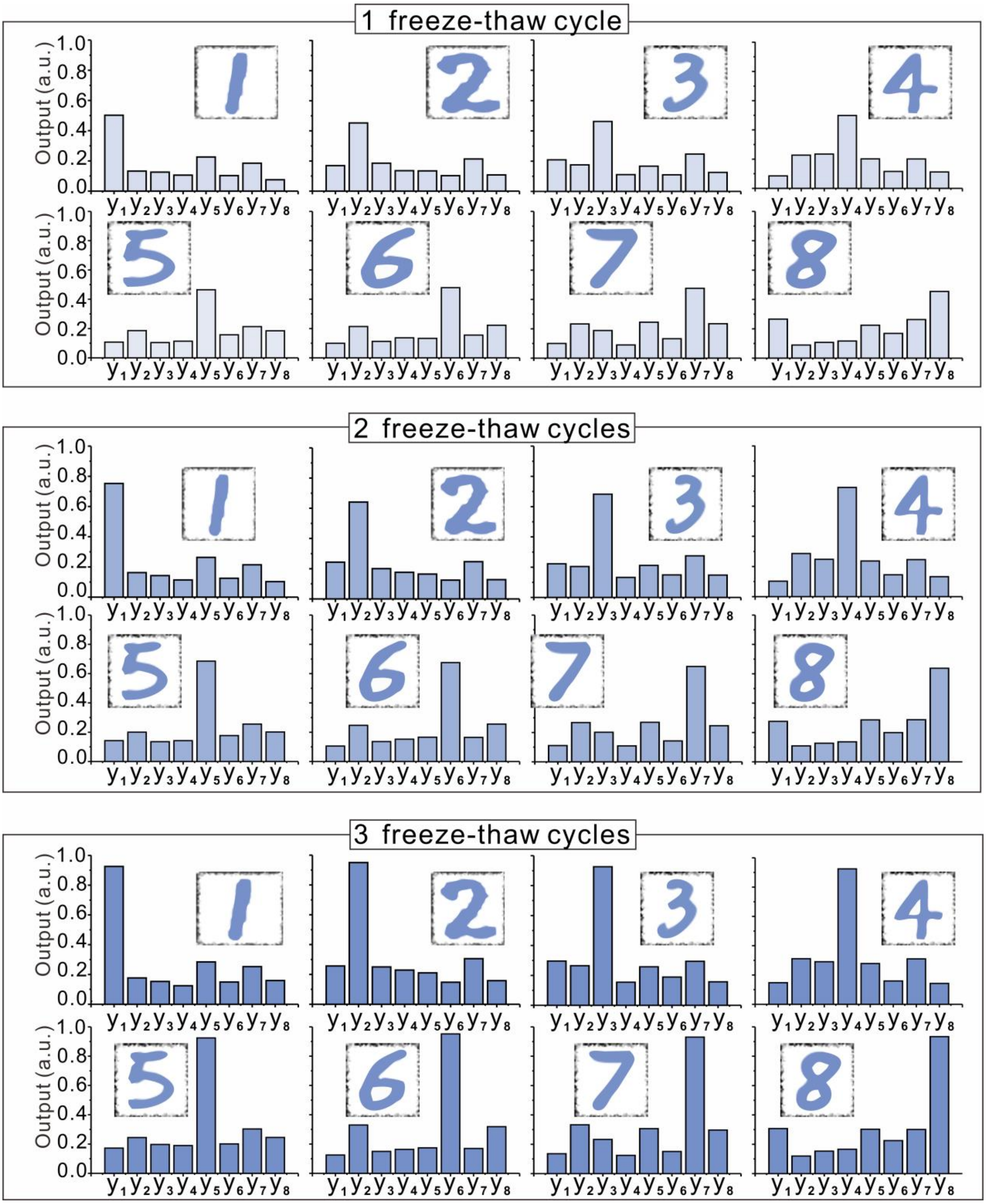

Fig. S12. Repeated freeze-thaw cycles were used to accelerate DNA-based ConvNet circuit when using SO_4^{2-} as magnesium counterion.

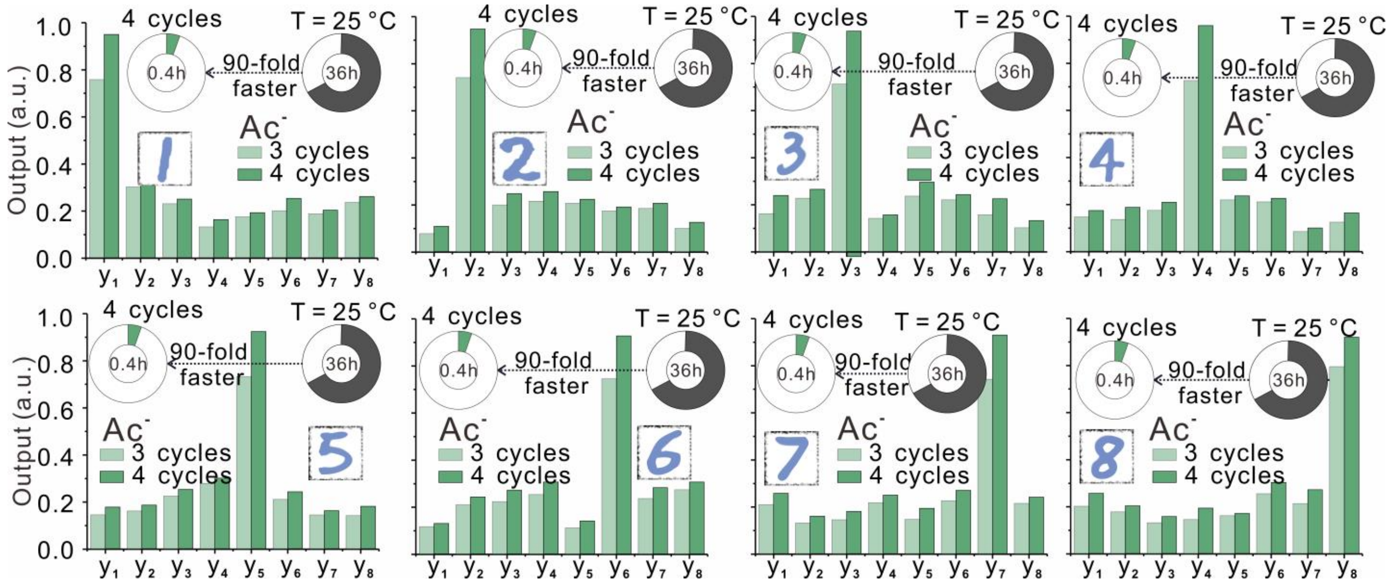

Fig. S13. Repeated freeze-thaw cycling speeds up large-scale DNA-based ConvNet when using Ac⁻ as magnesium counterion. Through repeated freeze-thaw cycling, we found that computation of this network circuit was accelerated, in which 4 cycles of freeze-thaw allowed each input patterns to trigger the almost same output level as operating at 25 °C. The numbers in the rings represented the time required to complete the pattern recognition with the repeated freeze-thaw cycling or operation at 25 °C. Note that when negative counterions were changed, we could barely observe detectable changes in behavior of circuit operated at 25 °C. Experiments were conducted in TE (pH 8.0) buffer containing 12.5 mM $(CH_3COO)_2Mg$ (freeze-thaw cycling) or $MgSO_4$ (operation at 25 °C).

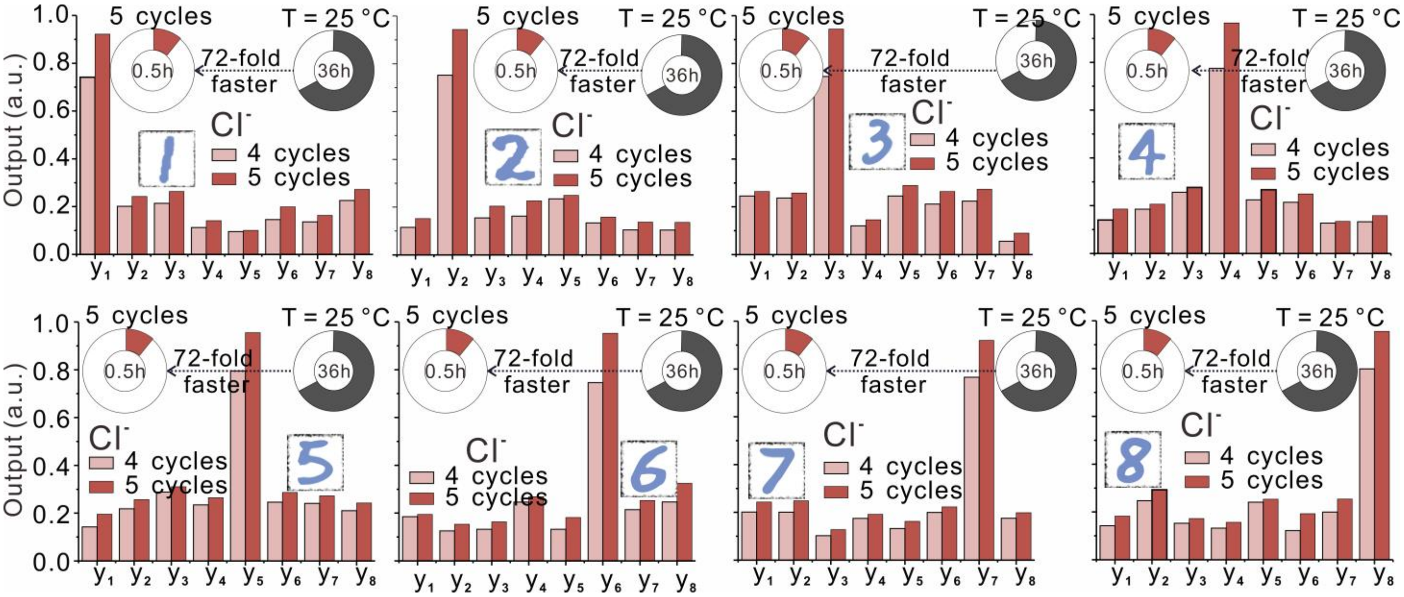

Fig. S14. Repeated freeze-thaw cycling speeds up large-scale DNA-based ConvNet when using Cl^- as magnesium counterion. Through repeated freeze-thaw cycling, we found that computation of this network circuit was accelerated, in which 5 cycles of freeze-thaw allowed each input patterns to trigger the almost same output level as operating at 25 °C. The numbers in the rings represented the time required to complete the pattern recognition with the repeated freeze-thaw cycling or operation at 25 °C. Note that when negative counterions were changed, we could barely observe detectable changes in behavior of circuit operated at 25 °C. Experiments were conducted in TE (pH 8.0) buffer containing 12.5 mM $MgCl_2$ (freeze-thaw cycling) or $MgSO_4$ (operation at 25 °C).

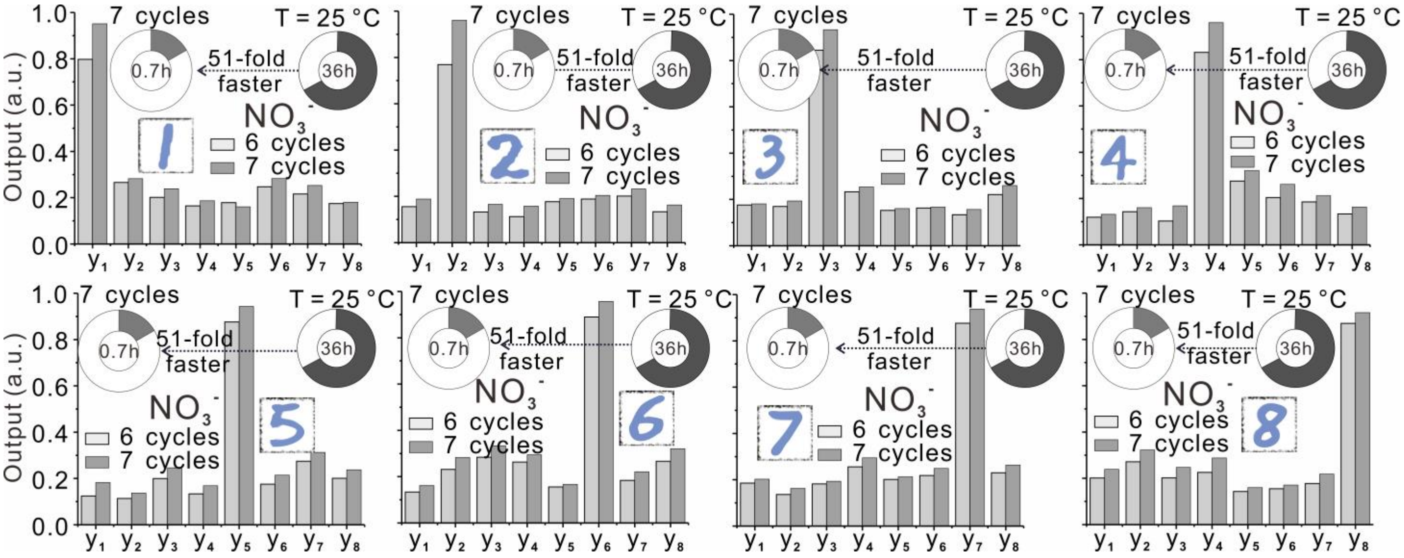

Fig. S15. Repeated freeze-thaw cycling speeds up large-scale DNA-based ConvNet when using NO_3^- as magnesium counterion. Through repeated freeze-thaw cycling, we found that computation of this network circuit was accelerated, in which 7 cycles of freeze-thaw allowed each input patterns to trigger the almost same output level as operating at 25 °C. The numbers in the rings represented the time required to complete the pattern recognition with the repeated freeze-thaw cycling or operation at 25 °C. Note that when negative counterions were changed, we could barely observe detectable changes in behavior of circuit operated at 25 °C. Experiments were conducted in TE (pH 8.0) buffer containing 12.5 mM $MgCl_2$ (freeze-thaw cycling) or $MgSO_4$ (operation at 25 °C).

Table S1. Comparison of reaction times required for strand displacement reactions.

Strand displacement reactions	Number of DNA species	Freeze-thaw cycling	Operation at 25 ℃	Acceleration effect
		SO_4^{2-}	SO_4^{2-}	SO_4^{2-}
toehold = 4 nt	2	8 cycles (0.8 h)	20 h	25-fold
toehold = 3 nt	2	10 cycles (1.0 h)	20 h	20-fold
toehold = 2 nt	2	10 cycles (1.0 h)	20 h	20-fold

Table S2. Comparison of computation times required for DNA circuits

Circuits	Number of DNA species (excluding input strands)	Freeze-thaw cycling				Operation at 25 °C	Acceleration effect
		SO_4^{2-}	Ac^{-}	Cl^{-}	NO_3^{-}	SO_4^{2-}	SO_4^{2-} (Compared with operation at 25 °C)
1-layer (OR)	6	5 cycles (0.5 h)	6 cycles (0.6 h)	7 cycles (0.7 h)	9 cycles (0.9 h)	4 h	8-fold
1-layer (AND)	7	5 cycles (0.5 h)	7 cycles (0.7 h)	10 cycles (1.0 h)	14 cycles (1.4 h)	16 h	32-fold
2-layer (AND-OR)	13	6 cycles (0.6 h)	9 cycles (0.9 h)	12 cycles (1.2 h)	16 cycles (1.6 h)	10 h	17-fold
3-layer (OR-AND-OR)	19	7 cycles (0.7 h)	11 cycles (1.1 h)	15 cycles (1.5 h)	18 cycles (1.8 h)	12 h	17-fold
DNA ConvNet	~200	3 cycles (0.3 h)	4 cycles (0.4 h)	5 cycles (0.5 h)	7 cycles (0.7 h)	36 h	120-fold

Table S3.

All DNA sequences used in this work.
